# Editorial for the Special Issue on MEMS Mirrors

**DOI:** 10.3390/mi9030099

**Published:** 2018-02-27

**Authors:** Huikai Xie

**Affiliations:** Department of Electrical and Computer Engineering, University of Florida, Gainesville, FL 32611, USA; hkx@ufl.edu

MEMS mirrors can steer, modulate, and switch light, as well as control the wavefront for focusing or phase modulation. MEMS mirrors have found enormous commercial success in projectors, displays, and fiberoptic communications. Micro-spectrometers based on MEMS mirrors are starting to appear in the consumer market as well. There are also many breakthroughs in applying MEMS mirrors for endoscopic imaging. There is a new wave of opportunities for MEMS mirrors coming up, such as the micro-LiDAR for autonomous driving and robotics, optical cross-connect (OXC) for cloud data centers, and optical scanners for virtual reality/augmented reality. Of course, there are numerous challenges that researchers and engineers must overcome to fully utilize the potential of MEMS mirrors. For example, modeling and control are inherently complex due to the multiphysics, multi-DOF, and nonlinear nature of the micro-actuators for MEMS mirrors. Reliability is always a huge hurdle for commercialization and the tradeoffs among the speed, aperture, and scan range are often overwhelming. 

There are 15 papers published in this special issue, covering MEMS mirrors based on all of the commonly used actuation mechanisms including electrostatic [1,2,3,4,5], electromagnetic [6,7,8], piezoelectric [9], and electrothermal [10,11,12,13,14]. Half of these papers explore various aspects of MEMS mirrors, such as working in harsh environments [1], spring hardening compensation [3], optimal packaging conditions for resonance operation [5], input saturation control [8], extremely large scan angle [9], overshoot suppression [10], electrothermal actuator modeling [13], and design optimization [14]. There is also a paper reporting a passive micromirror [15]. The other half of the papers are focused on a range of applications of MEMS mirrors including grayscale photolithography [2], biomedical imaging [4,11,12], laser display [6], and LiDAR [7]. 

In particular, Zamkotsian et al. tested a deformable mirror (DM) array of single crystalline silicon hexagonal tip-tilt-piston mirrors in a cryo-vacuum chamber designed for reaching 10^−6^ mbar and 160 K. The DM array survived, which provides a viable solution for DM arrays in harsh environments such as in space [1]. Izawa et al. applied the spring softening effect of electrostatic comb drives to compensate for the spring hardening effect of torsion-bar springs [3]. Zhao et al. identified an optimal range of vacuum for operating electrostatic comb-drive micromirrors [5]. Tan et al. proposed a control design framework based on composite nonlinear feedback (CNF) and the integral sliding mode (ISM) technique to improve MEMS micromirrors’ performance under input saturation [8]. Gu-Stoppel et al. presented a lead zirconate titanate (PZT)-actuated micromirror that achieves an extremely large scan angle of up to 106° and a high frequency of 45 kHz simultaneously [9]. M. Li et al. effectively eliminated the overshoot and oscillation of electrothermally-actuated micromirrors simply by setting the product of the thermal response time and the fundamental resonance frequency to be greater than Q/2π [10]. Torres et al. reported the modeling of a MEMS mirror structure with four actuators driven by the phase-change of VO_2_ thin film [13]. Saleem et al. presented the parametric design optimization of an electrothermally actuated micromirror for the deflection angle, input power, and micromirror temperature rise from ambient conditions [14]. Sabry et al. fabricated a silicon micromachined three-dimensional curved mirror for optical fiber light collimation [15]. 

On the applications side, Deng et al. successfully demonstrated maskless grayscale photolithography using Texas Instruments’ digital micromirror devices (DMDs) [2]. H. Li et al. employed a novel lever-based compliant mechanism to enable large vertical displacements of a reflective mirror for the axial scanning of a multi-photon fluorescence imaging microscopy system [4]. Li et al. fabricated a two-axis electromagnetic micromirror with Ni electroplated on the mirror plate to eliminate Joule heating and applied the micromirror to a laser display system, effectively reducing laser speckles [6]. Ye et al. fabricated a Ti-alloy-based electromagnetic micromirror with a very large aperture of 12 mm and a rapid scanning frequency of 1.24 kHz using electrical discharging and explored its potential application for micro-LiDAR [7]. Lara-Castro et al. proposed a vertical scanning electrothermal bimorph micromirror design by employing polysilicon as the heater material [11]. Tanguy et al. demonstrated a two-axis micromirror with a pair of electrothermal actuators and a set of passive torsion bars and applied it to an ultra-compact Mirau interferometer-based optical coherence tomography (OCT) imaging probe [12]. 

I would like to take this opportunity to thank all the authors for submitting their papers to this special issue. I also want to thank all the reviewers for dedicating their time and helping to improve the quality of the submitted papers. 
